# New Curculionoidea (Coleoptera) records for Quebec, Canada

**DOI:** 10.3897/zookeys.681.12469

**Published:** 2017-06-21

**Authors:** Pierre de Tonnancour, Robert S. Anderson, Patrice Bouchard, Claude Chantal, Stéphane Dumont, Robert Vigneault

**Affiliations:** 1 22 5e avenue, Terrasse-Vaudreuil, Quebec, Canada, J7V 3P5; 2 Canadian Museum of Nature, P.O. Box 3443, Station D, Ottawa, Ontario, Canada, K1P 6P4; 3 Canadian National Collection of Insects, Arachnids and Nematodes, Agriculture and Agri-Food Canada, 960 Carling Avenue, Ottawa, Ontario, Canada, K1A 0C6; 4 302 Gabrielle-Roy, Varennes, Quebec, Canada, J3X 1L8; 5 Département de biologie et de biotechnologies, Collège Ahuntsic, 9155 rue Saint-Hubert, Montréal, Quebec, Canada, H2M 1Y8; 6 16 rue du Mont Saint-Pierre, Oka, Quebec, Canada, J0N 1E0

**Keywords:** Curculionoidea, Anthribidae, Brentidae, Curculionidae, new records, Canada, Quebec, Ontario, weevils

## Abstract

The following species of Curculionoidea are newly recorded from the Canadian province of Quebec: *Coelocephalapion emaciipes* (Fall, 1898); *Ischnopterapion virens* (Herbst, 1797); *Omphalapion hookerorum* (Kirby, 1808); *Perapion punctinasum* (J.B. Smith, 1884) (all Brentidae); *Anthonomus robustulus* LeConte, 1876; *Pseudanthonomus helvolus* (Boheman, 1843); *Bagous magister* LeConte, 1876; *Bagous tanneri* O’Brien, 1979; *Buchananius striatus* (LeConte, 1876); *Ceutorhynchus bolteri* Dietz, 1896; *Ceutorhynchus pallidactylus* (Marsham, 1802); *Ceutorhynchus pauxillus* Dietz, 1896; *Conotrachelus buchanani* Schoof, 1942; *Conotrachelus pusillus* LeConte, 1878; *Conotrachelus recessus* (Casey, 1910); *Curculio rubidus* (Gyllenhal, 1835); *Cylindrocopturus longulus* (LeConte, 1876); *Hadroplontus litura* (Fabricius, 1775); *Hypera rumicis* (Linnaeus, 1758); *Lixus terminalis* LeConte, 1876; *Myosides seriehispidus* Roelofs, 1873; *Phloeotribus dentifrons* (Blackman, 1921); *Plocamus echidna* (LeConte, 1876); *Scolytus muticus* Say, 1824; *Sirocalodes sericans* (LeConte, 1876); *Smicronyx sculpticollis* Casey, 1892 (all Curculionidae). Among these, *Buchananius striatus*, *Conotrachelus buchanani*, *Conotrachelus pusillus*, and *Curculio rubidus* (all Curculionidae) are also recorded from Canada for the first time. The latter is also newly reported from Ontario. Collecting data are provided for *Lixus punctinasus* LeConte, 1876, previously reported to occur in Canada without any further information, and for *Choragus sayi* LeConte, 1876 (Anthribidae) and *Rhyssomatus aequalis* Horn, 1873 (Curculionidae), both previously recorded from Quebec, also without further details.

## Abstract (fr)

Les auteurs mentionnent pour la première fois la présence au Québec des espèces de Curculionoidea suivantes : *Coelocephalapion emaciipes* (Fall, 1898); *Ischnopterapion virens* (Herbst, 1797); *Omphalapion hookerorum* (Kirby, 1808); *Perapion punctinasum* (J.B. Smith, 1884) (tous des Brentidae); *Anthonomus robustulus* LeConte, 1876; *Pseudanthonomus helvolus* (Boheman, 1843); *Bagous magister* LeConte, 1876; *Bagous tanneri* O’Brien, 1979; *Buchananius striatus* (LeConte, 1876); *Ceutorhynchus bolteri* Dietz, 1896; *Ceutorhynchus pallidactylus* (Marsham, 1802); *Ceutorhynchus pauxillus* Dietz, 1896; *Conotrachelus buchanani* Schoof, 1942; *Conotrachelus pusillus* LeConte, 1878; *Conotrachelus recessus* (Casey, 1910); *Curculio rubidus* (Gyllenhal, 1835); *Cylindrocopturus longulus* (LeConte, 1876); *Hadroplontus litura* (Fabricius, 1775); *Hypera rumicis* (Linnaeus, 1758); *Lixus terminalis* LeConte, 1876; *Myosides seriehispidus* Roelofs, 1873; *Phloeotribus dentifrons* (Blackman, 1921); *Plocamus echidna* (LeConte, 1876); *Scolytus muticus* Say, 1824; *Sirocalodes sericans* (LeConte, 1876); *Smicronyx sculpticollis* Casey, 1892 (tous des Curculionidae). Parmi ces espèces, *Buchananius striatus*, *Conotrachelus buchanani*, *Conotrachelus pusillus* et *Curculio rubidus* (tous des Curculionidae) sont également mentionnés pour la première fois comme étant présents au Canada et, dans le cas de la dernière espèce, également en Ontario. Des données de collecte sont fournies pour *Lixus punctinasus* LeConte, 1876, déjà répertorié au Canada sans données de capture, ainsi que pour *Choragus sayi* LeConte, 1876 (Anthribidae) et *Rhyssomatus aequalis* Horn, 1873 (Curculionidae), dont la présence avait déjà été signalée au Québec, également sans données de capture.

## Introduction

Douglas et al. (2013) recently reported five Brentidae and 29 Curculionidae species new to Quebec (of which 3 and 11 were new to Canada, respectively), increasing the total number of species of each family known to occur in the province to 22 and 386, respectively (Bousquet et al. 2013). Recent collection efforts, mainly by amateur entomologists, have since yielded new findings. The new records and new documented record (*Lixus punctinasus* LeConte, 1876) reported herein (4 Brentidae and 23 Curculionidae), listed according to the classification of Bouchard et al. (2011), bring these totals to 26 and 409, respectively. Additions to the province’s weevil fauna will undoubtedly be recorded in the years to come not only due to increased collection efforts, but as more species continue to expand their range northwardly under present global warming conditions or are being introduced from other countries. Among the species newly reported here, *Ischnopterapion virens* (Herbst, 1797), *Omphalapion hookerorum* (Kirby, 1808), *Ceutorhynchus pallidactylus* (Marsham, 1802), *Curculio rubidus* (Gyllenhal, 1835), *Hadroplontus litura* (Fabricius, 1775), *Myosides seriehispidus* Roelofs, 1873, and *Hypera rumicis* (Linnaeus, 1758) are all adventive species (*sensu* Wheeler and Hoebeke 2009) that were accidentally introduced in Canada or intentionally brought in as biological control agents.

## Materials and methods

Specimens belonging to species recorded or referred to in the present article were identified (or their identity was confirmed) by recognized specialists listed henceforth under each species name by their name, or if an author of this paper, by their initials.

Label data are provided in chronological order for every species. These data were translated from French to English, and various details (e.g., current regional county municipality [MRC], collecting technique, general habitat), when known, have been added between brackets.

Specimens were either swept or beaten from various plant species, attracted to mercury vapour, ultraviolet or porch lights or handpicked from various substrates or from a flight interception trap made of tulle fabric (~2,5m x 10m) held between two wood piles or set up in a suburban backyard.

Plant family, generic and specific names follow the classification used in Database of Vascular Plants of Canada (VASCAN) (http://data.canadensys.net/vascan/search).

Acronyms of collections referred to in this article are as follows:

**CCCH** Claude Chantal Insect Collection (private collection), Varennes, Quebec, Canada

**CCOB** Charles W. O’Brien Insect Collection (private collection), Green Valley, Arizona, United States

**CCTE** Claude Tessier Insect Collection (private collection), Quebec, Quebec, Canada

**CHMS** Henri Miquet-Sage Insect Collection (private collection), Mont-Saint-Hilaire, Quebec, Canada

**CMNC** Canadian Museum of Nature, Ottawa, Ontario, Canada

**CNCI** Canadian National Collection of Insects, Arachnids, and Nematodes, Agriculture and Agri-Food Canada Research Centre, Ottawa, Ontario, Canada

**CPTO** Pierre de Tonnancour Insect Collection (private collection), Terrasse-Vaudreuil, Quebec, Canada

**CRVI** Robert Vigneault Insect Collection (private collection), Oka, Quebec, Canada

**CSDU** Stéphane Dumont Insect Collection (private collection), Montreal, Quebec, Canada

**CSLA** Serge Laplante Insect Collection (private collection), Gatineau, Quebec, Canada

## Results

### Family Anthribidae Billberg, 1820

#### Subfamily Choraginae Kirby, 1819

##### Tribe Choragini Kirby, 1819

###### Choragus sayi

Taxon classificationAnimaliaColeopteraAnthribidae

LeConte, 1876, new data supporting first record for Quebec

Species identification confirmed by RSA, 2015 and 2016

####### Note.

Bousquet et al. (2013) recorded this species from Quebec without any further comment, possibly on the basis of a vague record (“Quebec to Georgia west to Indiana and Texas”) by Valentine (1998). This small species is strongly saltatorial and can be difficult to catch in hot weather. We provide, for the first time, data on the occurrence of this species in the province.

####### Specimen data.

[Agglomération de Longueuil] Longueuil, 18VII1992, C. Chantal (1, CCCH); [MRC Marguerite-d’Youville] Varennes, 16VII1999, attracted to UV light, C. Chantal (1, CCCH); same except: 29VI2006 (1, CCCH); [MRC Brome-Missisquoi] Saint-Armand, 2VIII2007, understory, on foliage, C. Chantal (1, CCCH); [MRC Marguerite-D’Youville] Île Sainte-Thérèse, 1IX2009, C. Chantal (1, CCCH); [MRC Deux-Montagnes] Parc national d’Oka, La Grande Baie, 19VII2014, beaten from dead branches over forest litter, R. Vigneault (3, CRVI); [MRC Coaticook] Compton, 25VIII2014, C. Levesque (1, CNCI); [MRC Deux-Montagnes] Parc national d’Oka, La Grande Baie, 27VI2015, beaten from dead branches over forest litter, R. Vigneault (1, CRVI); same except: 30VI2015 (16:00), P. de Tonnancour (1, CPTO); same except: 2VII2015, R. Vigneault (1, CRVI); same except: 5VII2015 (16:00), beaten from dead branches of *Acer saccharum*, P. de Tonnancour & R. Vigneault (1, CMNC; 4, CPTO; 6, CRVI); same except: 9VII2015, R. Vigneault (2, CRVI); [MRC Deux-Montagnes] Parc national d’Oka, Calvaire, 25VI2016, beaten from dead branches over forest litter, R. Vigneault (3, CRVI); same except: 1VII2016 (19, CRVI); same except: La Grande Baie, 6VII2016, beaten from dead branches of *Acer saccharum*, R. Vigneault (11, CPTO); same except: La Grande Baie, 6VII2016, beaten from dead branches over forest litter, R. Vigneault (1, CRVI), 12VII2016 (1, CRVI), and 1VIII2016 (1, CRVI).

### Family Brentidae Billberg, 1820

#### Subfamily Apioninae Schönherr, 1823

##### Tribe Apionini Schönherr, 1823

###### Perapion punctinasum

Taxon classificationAnimaliaColeopteraBrentidae

(J.B. Smith, 1884), new to Quebec

Species identification confirmed by RSA, 2016

####### Note.

This native species is easily separated from all other Apioninae known to occur in Quebec by the conspicuous elongate postscutellar spot of white vestiture and spot of dense white scales at the base of elytral interstriae 2 and 3. Nothing is known of its habits or life history, except that adults were collected in August on dock, *Rumex* L. spp., including golden dock, *Rumex persicarioides* L. (Polygonaceae) (Bright 1993). Ontario was until now considered as the eastward limit of its range in Canada (Bousquet et al. 2013). A photograph of one of the specimens reported herein is posted on bugguide.net (http://bugguide.net/node/view/1077586/bgpage).

####### Specimen data.

[MRC Deux-Montagnes] Parc national d’Oka, composting site, 29V2015, white tulle fabric flight interception trap, R. Vigneault (2, CRVI).

###### Omphalapion hookerorum

Taxon classificationAnimaliaColeopteraBrentidae

(Kirby, 1808), new to Quebec

Species identification confirmed by RSA, 2015

####### Note.

This Palaearctic adventive species was recorded for the first time in North America in 1993 based on specimens collected in Nova Scotia in 1990 (Peschken 1993 and Sampson and MacSween 1993, *in* Majka et al. 2007b). In Canada, it was subsequently released and is established as a biological control agent against scentless chamomile, *Tripleurospermum inodorum* (L.) Sch.Bip. (= *Matricaria perforata* Mérat) (Asteraceae) in British Columbia, Alberta, Saskatchewan, and Manitoba (McClay and De Clerck-Floate 1999). It was also collected on stinking chamomile, *Anthemis cotula* L. (Asteraceae), in Nova Scotia (Majka et al. 2007b).

####### Specimen data.

[MRC La ValléeduRichelieu] Saint-Charles-sur-Richelieu, 29VI2003, H. Miquet-Sage (3, CHMS; 1, CPTO); [MRC Marguerite-D’Youville] Varennes, 30VI2008, C. Chantal (2, CCCH); [MRC La ValléeduRichelieu] Mont-Saint-Hilaire, 2VII2008, H. Miquet-Sage (2, CHMS); [MRC Marguerite-D’Youville] Varennes, 2V2010, C. Chantal (2, CCCH; 1, CPTO); same except: 20V2010 (1, CCCH), and 9VI2010 (1, CCCH); [MRC La ValléeduRichelieu] MontSaintHilaire, 13VI2010, H. Miquet-Sage (1, CPTO); [MRC Marguerite-D’Youville] Varennes, 30V2011, C. Chantal (1, CCCH); same except 9VI2012 (1, CCCH), and 21V2014 (1, CCCH); [MRC La ValléeduRichelieu] MontSaintHilaire, 12V2014, H. MiquetSage (1, CHMS); same except: 20VI2014 (1, CHMS), and 25VI2014 (1, CHMS); [MRC Marguerite-D’Youville] Varennes, 7VI2015, C. Chantal (1, CCCH).

###### Ischnopterapion virens

Taxon classificationAnimaliaColeopteraBrentidae

(Herbst, 1797), new to Quebec

Species identification confirmed by RSA, 2016

####### Note.

Widely distributed through most of the Palaearctic region (Alonso-Zarazaga 2011), this adventive species was recorded for the first time in North America in 1994, in Pennsylvania (Hoebeke et al. 2000). Until now, it was known to occur in Canada only in the Maritime Provinces (Bousquet et al. 2013). It is considered a pest of clovers, *Trifolium* L. spp. (Fabaceae). It can be distinguished from the superficially similar *Stenopterapion meliloti* Kirby, 1808, by its smaller size and the bluish colour of its pronotum and venter (black in *S. meliloti*). As indicated by Hoebeke et al. (2000) and by the label data provided hereafter, the flight season extends until late in the year.

####### Specimen data.

[MRC Haut-Richelieu] Henryville [dike adjacent to Réserve écologique Marcel-Raymond], 29IX2012, C. Chantal (2, CCCH); same except: 3X2013 (3, CCCH); MRC Vaudreuil-Soulanges, Terrasse-Vaudreuil, 15IX2014 (15:00), white tulle fabric flight interception trap, P. de Tonnancour (1, CPTO); same except: 7X2014 (15:00) (1, CPTO) and 12X2014 (17:00) (1, CPTO); [MRC Coaticook] Waterville, 11VII2015, H. Miquet-Sage (1, CHMS); MRC Vaudreuil-Soulanges, Terrasse-Vaudreuil, 21IX2015 (12:30), white tulle fabric flight interception trap, P. de Tonnancour (1, CPTO); [MRC Marguerite-D’Youville] Varennes, 21IX2015, C. Chantal (1, CCCH); MRC Vaudreuil-Soulanges, Terrasse-Vaudreuil, 22IX2015 (15:00), white tulle fabric flight interception trap, P. de Tonnancour (1, CPTO); same except, 22IX2015 (15:00), beaten from *Oidium* infected foliage of *Helianthus strumosus*, (1, CPTO); same except: 27IX2015 (11:30, 13:30), white tulle fabric flight interception trap (2, CPTO); same except: 6X2015 (16:15) (1, CPTO), and 7X2015 (14:30–15:30) (3, CPTO); same except: 11X2015 (15:00), beaten from *Oidium* infested foliage of *Helianthus strumosus* (1, CPTO); same except: 12X2015 (11:00–15:00), white tulle fabric flight interception trap (8, CPTO; 2, CSDU); same except: 5XI2015 (14:00–15:00), climbing on pale house exterior wall (1, CPTO; 1, CRVI); same except: 6XI2015 (15:00) (1, CPTO), 9XI2015 (15:00) (1, CPTO), 19XI2015 (12:00) (1, CPTO), 26XI2015 (13:00) (1, CPTO), 27XI2015 (12:30) (1, CCCH), 11XII2015 (13:00–15:00) (4, CPTO), and 12XII2015 (12:00) (1, CPTO); Montreal, Parc Zotique-Racicot (45.5427, -73.6903), 11V2106, swept from *Trifolium* sp., S. Dumont (6, CSDU); same except: 12-V-2016 (3, CSDU); MRC Haut-Richelieu, Henryville, dike adjacent to Réserve écologique Marcel-Raymond], 12V2016 (13:00–16:00), swept from grasses, *Equisetum* and *Solidago*, P. de Tonnancour (1, CPTO); MRC Vaudreuil-Soulanges, Notre-Dame-de-l’Île-Perrot, 20V2016 (17:00), swept from *Trifolium pratense*, P. de Tonnancour (11, CPTO); Montreal, Parc Zotique-Racicot (45.5427, -73.6903), 23V2106, swept from *Trifolium* sp., S. Dumont (5, CSDU); same except: 24V2016 (2, CSDU); MRC Brome-Missisquoi, SaintArmand, 25V2016 (16:00), swept from *Trifolium pratense*, P. de Tonnancour (6, CPTO); MRC Vaudreuil-Soulanges, Saint-Lazare, 29VI2013 (16:00–17:00), swept from *Trifolium pratense*, P. de Tonnancour (2, CPTO); MRC Laval, Laval, rue des Charmes (45.5884, -73.8244), 20VII2016 (15:00), swept from *Trifolium pratense*, P. de Tonnancour (2, CPTO); MRC Vaudreuil-Soulanges, Terrasse-Vaudreuil, 10XI2016 (15:00), climbing on pale house exterior wall (1, CPTO).

###### Coelocephalapion emaciipes

Taxon classificationAnimaliaColeopteraBrentidae

(Fall, 1898), new to Quebec

Species identification confirmed by RSA, 2016

####### Note.

The occurrence of this small native pale-legged species in the province was expected as it was previously known in Canada from Ontario and the Maritime Provinces. Although this species has been tentatively associated with tick-trefoil, *Desmodium* Desv. sp. (Fabaceae), based on the very few available data at the time (Bright 1993), it is worth noting that most of the specimens caught in 2016 were found in association with *Scirpus* L. spp. (Cyperaceae) in wet habitats.

####### Specimen data.

[MRC Marguerite-D’Youville] Varennes, 30VI2014, C. Chantal (1, CCCH); same except: 15V2015 (1, CCCH); MRC Vaudreuil-Soulanges, Notre-Dame-de-l’Île-Perrot, 11V2016 (13:00), swept from *Scirpus atrovirens* (1, CPTO); same except: 14V2016 (15:00) (1, CPTO); [MRC Deux-Montagnes] Parc national d’Oka, 19V2016, swept from herbs in field (2, CRVI); MRC Brome-Missisquoi, SaintArmand, 25V2016 (15:00), swept from *Scirpus* sp. (2, CPTO).

### Family Curculionidae Latreille, 1802

#### Subfamily Curculioninae Latreille, 1802

##### Tribe Anthonomini C.G. Thompson, 1859

###### Anthonomus robustulus

Taxon classificationAnimaliaColeopteraCurculionidae

LeConte, 1876, new to Quebec

Species identification confirmed by RSA, 2015

####### Note.

This small native species is characterized by its 6-jointed funicle, compact, short and broad form, and light bluish-gray scales. It is said to occur on goldenrods, *Solidago* L. spp. (Asteraceae) (Blatchley and Leng 1916). It was previously known in Canada from Alberta, Saskatchewan, and New Brunswick (Bousquet et al. 2013).

####### Specimen data.

[MRC Brome-Missisquoi] Saint-Armand, 7VI2004, C. Chantal (2, CCCH); same except: 3VII2006 (2, CCCH), and 2VII2008 (2, CCCH); MRC Vaudreuil-Soulanges, Notre-Dame-de-l’Île-Perrot, 31V2011 (13:00), meadow, swept from *Solidago*/*Aster*, P. de Tonnancour (2, CPTO); same except: 1VI2011 (14:00) (1, CPTO); [MRC Brome-Missisquoi] Saint-Armand, 3VIII2011, C. Chantal (1, CCCH); [MRC Haut-Richelieu] Henryville [dike adjacent to Réserve écologique Marcel-Raymond], 28V2013, (14:00–17:00), swept from grasses, *Equisetum* and *Solidago*, C. Chantal and P. de Tonnancour (1, CCCH; 1, CPTO); MRC HautSaintLaurent, Saint-Anicet (45°0422, 74.4473), 14VI2013 (18:00), beaten from *Cornus stolonifera*, P. de Tonnancour (1, CPTO); same except: 15VI2013 (13:00), wet meadow, swept from various herbaceous plants, P. de Tonnancour (1, CPTO); [MRC La ValléeduRichelieu] Mont-Saint-Hilaire, 24VI2013, H. Miquet-Sage (1, CHMS); MRC Haut-Saint-Laurent, Franklin, roadside opposite to Réserve écologique du Pin-Rigide, 17VII2013 (14:00), beaten from *Lythrum salicaria*, P. de Tonnancour (7, CPTO); MRC Haut-Richelieu, Henryville, dike adjacent to Réserve écologique Marcel-Raymond, 8VI2014 (14:00–16:00), swept from grasses, *Equisetum* and *Solidago*, P. de Tonnancour (4, CPTO); [MRC Brome-Missisquoi] Saint-Armand, 16VI2014, C. Chantal (1, CCCH); MRC Haut-Richelieu, Henryville, dike adjacent to Réserve écologique Marcel-Raymond, 4VI2015, P. de Tonnancour (16:00–18:00) (1, CPTO); [MRC Brome-Missisquoi] Saint-Armand (45.0199, -73.0838), 25V2016, S. Dumont (1, CSDU).

###### Pseudanthonomus helvolus

Taxon classificationAnimaliaColeopteraCurculionidae

(Boheman, 1843), new to Quebec

Species identification confirmed by RSA, 2016

####### Note.

This native species is associated with witch hazel, *Hamamelis virginiana* L. (Hamamelidaceae) (Clark 1987). Adults emerging from hibernation are active from mid-May to early July, and those from the current-year generation emerge from mid-August to early September and then hibernate until the following spring (DeSteven 1981).

####### Specimen data.

MRC Deux-Montagnes, Parc national d’Oka (45.4916, 74.0137), 30VI2015 (17:00), beaten from *Hamamelis virginiana*, P. de Tonnancour & R. Vigneault (4, CPTO; 5, CRVI); same except: 2VII2015 (18:00) (16, CPTO) and 5VII2015 (18:00) (1, CCCH; 6, CPTO); same except: 5IX2015 (17:00), R. Vigneault (1, CPTO) and 20VIII2016 (2, CRVI); same except: 27-VIII-2016 (14:00), P. de Tonnancour (2, CNCI; 2 CMNC; 13, CPTO; 1, CSDU); same except: (45.4619, 74.0489), 27VIII2016 (16:00) (1, CPTO; 3, CRVI).

##### Tribe Curculionini Latreille, 1802

###### Curculio rubidus

Taxon classificationAnimaliaColeopteraCurculionidae

(Gyllenhal, 1835), new to Canada

Species identification confirmed by RSA, 2015

####### Note.

This record comes three years after the first North American detection of the species in Michigan (Anderson et al. 2013) and is based on the incidental finding in the Montreal area (Notre-Dame-de l’Île-Perrot) of a single specimen in 2013 and of dozens of additional specimens 2–3 years later. This widespread Palaearctic species is easily separated from all North American native *Curculio* species by its very small size (<3.5mm), lack of femoral teeth and association with birch, *Betula* L. spp. All specimens recorded in Quebec were collected in a stand of gray birch, *Betula populifolia* Marshall (Betulaceae), and most were directly beaten from gray birch. Adults are said to be active from May to October in Europe (Hoffmann 1954; as “*Balaninus undulatus* Herbst, 1795”), but all specimens reported herein were captured in August (except three specimens caught on September 1, 2016). This species is also newly recorded from Ontario, based on a specimen photographed by Burke Korol in Barrie, Simcoe County, on August 21, 2015 and posted on bugguide.net (http://bugguide.net/node/view/1127147).

####### Specimen data.

MRC Vaudreuil-Soulanges, Notre-Dame-de-l’Île-Perrot, 10VIII2013 (17:00), beaten from *Quercus rubra*, P. de Tonnancour (1, CPTO); same except: 8VIII2015 (15:00), beaten from *Alnus rugosa* (1, CPTO), beaten from *Betula populifolia* (2, CPTO) or swept from various herbaceous plants in gray birch stand (1, CPTO); same except: 10VIII2015 (13:00), swept from various herbaceous plants in gray birch stand (2, CPTO); same except: 16VIII2015 (15:00), beaten from *Betula populifolia* (4, CCOB; 5, CMNC; 5, CNCI; 18, CPTO; 1, CRVI); 17VIII2015 (14:00) (2, CNCI; 2, CPTO); 17VIII2015 (14:00 and 18:00) (2, CMNC; 2, CNCI; 11, CPTO); 18VIII2015 (19:00) (5, CNCI; 2, CSDU; 1, CRVI); 22VIII2015 (14:00), C. Chantal (10, CCCH); 20VIII2016 (16:00), P. de Tonnancour (3, CPTO); 23VIII2016 (16:00) (1, CPTO); 28VIII2016 (16:00) (2, CPTO); 29VIII2016 (18:00) (1, CPTO); 1IX2016 (13:00) (3, CPTO).

##### Tribe Smicronychini Seidlitz, 1891

###### Smicronyx sculpticollis

Taxon classificationAnimaliaColeopteraCurculionidae

Casey, 1892, new to Quebec

Species identification confirmed by RSA, 2015

####### Note.

This record is based on two specimens collected ten years apart from low vegetation in two localities. This native species was previously known in Canada only from Ontario (Bousquet et al. 2013). It is associated with dodders, *Cuscuta* L. spp. (Convolvulaceae) (Anderson 1962), obligate parasitic leafless vines almost entirely deprived of chlorophyll that wrap around various plants.

####### Specimen data.

[MRC Pierre-De Saurel] Saint-Roch-de-Richelieu, 20VI2005, C. Chantal (CCCH, 1); [MRC Deux-Montagnes] Parc national d’Oka, La Grande Baie, 30VI2015 [swept from low vegetation in swampy area], R. Vigneault (1, CRVI).

#### Subfamily Bagoinae C.G. Thompson, 1859

##### Bagous magister

Taxon classificationAnimaliaColeopteraCurculionidae

LeConte, 1876, new to Quebec

Species identification confirmed by RSA, 2016

###### Note.

This native species is easily separated from other species of its genus by the deep impressions on the prothorax, the elytral pattern created by fuscous and light brown scales with a fascia crossing the suture near middle, and its large size (Tanner 1943). It is associated with fragrant water-lily, *Nymphaea odorata* Aiton (Nymphaeaceae) (O’Brien et Marshall 1979). It was previously known in Canada only from Ontario (Bousquet et al. 2013).

###### Specimen data.

MRC Vaudreuil-Soulanges, Terrasse-Vaudreuil, 31V2013 (21:30), UV + porch light, P. de Tonnancour (2, CPTO); same except 30V2013 (1, CMNC); same except 23VI2013 (1, CMNC); MRC Deux-Montagnes, Parc national d’Oka, 30VII2012 (18:00), swept from Cyperaceae, *Polygonum* sp., *Pontederia cordata*, and *Sagittaria* sp., P. de Tonnancour (1, CPTO).

##### Bagous tanneri

Taxon classificationAnimaliaColeopteraCurculionidae

O’Brien, 1979, new to Quebec

Species identification confirmed by RSA, 2009, 2016

###### Note.

This native species was previously known in Canada only from Ontario (Bousquet et al. 2013). It feeds in the larval stage on submerged petioles of fragrant water-lily, *Nymphaea odorata* (McGaha 1952).

###### Specimen data.

[MRC Brome-Missisquoi] Saint-Armand, 2VII2001, attracted to UV lamp, C. Chantal (1, CCCH); [MRC Haut-Richelieu] Henryville, 24VI2003, attracted to UV lamp, C. Chantal (1, CCCH); [MRC Deux-Montagnes] Parc national d’Oka, La Grande Baie, 30VI2015 [swept from low vegetation, edge of swampy bay], R. Vigneault (1, CRVI); same except: 2VII2015 (16:00), beaten from dead branches, edge of swampy bay, P. de Tonnancour (1, CPTO).

#### Subfamily Baridinae Schönherr, 1836

##### Tribe Apostasimerini Schönherr, 1844

###### Buchananius striatus

Taxon classificationAnimaliaColeopteraCurculionidae

(LeConte, 1876), new to Canada

Species identification confirmed by RSA, 2015

####### Note.

This minute native species (1.4–1.6mm) is easily distinguished from all other Baridinae occurring in Quebec by its extremely wide and convex shape, its tiny size (genus *Buchananius* Kissinger, 1957, contains the smallest members of Nearctic Baridinae) and its vestiture of sparse but long erect scales. The only other North American congener, *Buchananius sulcatus* (LeConte, 1876), has been recorded as developing in the fruiting bodies of the fungus *Trichoderma peltatum* (Berk.) Samuels, Jaklitsch & Voglmayr (Hypocreaceae) growing on American Beech, *Fagus grandifolia* Ehrh. (Fagaceae), in Maryland (Prena et al. 2014). This species also represents a new record at the generic level for Canada.

####### Specimen data.

[MRC Joliette] Joliette, 7IX2013 [swept from forest understory], J.-F. Roch (1, CCCH); [MRC Deux-Montagnes] Parc national d’Oka, La Grande Baie, 28VI2014 [beaten/swept from undergrowth/fallen branches in deciduous stand], R. Vigneault (1, CRVI); same except: composting site, 28-V-2016 (19:00), white tulle fabric flight interception trap, R. Vigneault (1, CRVI); same except: Calvaire d’Oka, 1VII2016, beaten from fallen dead branches of deciduous tree, R. Vigneault (1, CRVI).

###### Plocamus echidna

Taxon classificationAnimaliaColeopteraCurculionidae

(LeConte, 1876), new to Quebec

Species identification confirmed by RSA, 2016

####### Note.

This remarkable native species was previously known in Canada only from Ontario (Bousquet et al. 2013). The circumstances under which all specimens caught in 2016 were found closely match the description provided by Drury (quoted by Blatchley and Leng 1916): “This curious little porcupine beetle was in clusters on trunk of a dead beech tree, near Cincinnati, Sept. 27, 1900. I took one cluster of 30; they very closely resemble the color of the bark”. A photograph of the 2015 specimen reported herein is posted on bugguide.net (http://bugguide.net/node/view/1078735/bgimage).

####### Specimen data.

[MRC Deux-Montagnes] Parc national d’Oka, composting site, 04VI2015 (18:00), white tulle fabric flight interception trap, R. Vigneault (1, CRVI); MRC Deux-Montagnes, Parc national d’Oka, La Grande Baie, 3VII2016, brushed from trunk of a recently dead *Fagus grandifolia*, R. Vigneault (18, CRVI); same except: 6VII2016 (12:00), P. de Tonnancour (1, CCCH; 1, CNCI; 16, CPTO; 1, CSDU); same except: 13VII2016 (17:00), P. de Tonnancour & R. Vigneault (1, CMNC; 4, CPTO, 4, CRVI); same except: 24VII2016 (17:00), P. de Tonnancour, R. Vigneault & S. Laplante (3, CPTO; 1, CRVI; 3, CSLA); same except: 1VIII2016, R. Vigneault (1, CRVI); same except: 20VIII2016 (2, CRVI).

#### Subfamily Ceutorhynchinae Gistel, 1848

##### Tribe Ceutorhynchini Gistel, 1848

###### Ceutorhynchus bolteri

Taxon classificationAnimaliaColeopteraCurculionidae

Dietz, 1896, new to Quebec

Species identification confirmed by RSA, 2014, 2016

####### Note.

This native species was previously recorded in Canada only from British Columbia (Bousquet et al. 2013), but it is known to occur in several states in eastern United States (O’Brien and Wibmer 1982). The British Columbia specimen (CNCI) was collected on spotted water-hemlock, *Cicuta occidentalis* Greene (now *Cicuta maculata* L.) (Apiaceae).

####### Specimen data.

[MRC Haut-Richelieu], Henryville, 28V2013, sweeping, C. Chantal (1, CCCH); MRC Deux-Montagnes, Parc national d’Oka, 29V2015 (18:00–20:00), white tulle fabric flight interception trap, P. de Tonnancour (1, CPTO); MRC Haut-Richelieu, Henryville, dike adjacent to Réserve écologique Marcel-Raymond, 4VI2015 (16:00–18:00), swept from grasses, *Equisetum* and *Solidago*, P. de Tonnancour (5, CPTO); same except: 12V2016 (13:00–16:00) (8, CPTO).

###### Ceutorhynchus pallidactylus

Taxon classificationAnimaliaColeopteraCurculionidae

(Marsham, 1802), new to Quebec

Species identification confirmed by PB, 2014

####### Note.

This adventive Palaearctic species, known as the cabbage stem weevil, is reported to attack several Brassicaceae and Resedaceae and is occasionally associated with *Cannabis sativa* L. (Cannabaceae) (Colonnelli 2004). It was previously known in Canada only from Nova Scotia (Bousquet et al. 2013), where it was first detected in 1994 (Majka et al. 2007a).

####### Specimen data.

[MRC Coaticook] Missisquoi Co., Mont le Pinacle, 10VI1984, Larochelle, Larivière (1, CNCI); [MRC Nouvelle-Beauce] East of St-Lambert-de-Lauzon, Rd. 218, 18VII2001, 46°36,133'N; 71°11.412'W, corn field with radish, Mason, Sarazin & Boudreault, QC 2001–110 (1, CNCI); same except: QC 2001–100 (1, CNCI); [MRC de l’Érable] NE of Plessisville, Road 116, 18VII2001, 46°18.796'N; 71°40.129'W, small canola field, Mason, Sarazin & Boudreault, QC 2001-330 (1, CNCI); [MRC Arthabaska] Saint-Albert, Hwy 122, 12VII2002, 46°00.455'N; 72°06.016'W, wild radish along edge of corn field, Mason, Boudreault & Farmakis, QC 2002-213 (1, CNCI); same except: QC 2002-214 (1, CNCI); [MRC Drummond] Domaine-Descoteaux, 22VII2003, 45°49.142'N; 72°13.983, J. Miall & P. Mason, wild mustard, QC03-121 (1, CNCI); [MRC Drummond] StGuillaume, 22VII2003, 45°54.909'N; 72°44.660'W, J. Miall & P. Mason, wild mustard, QC03-116 (1, CNC); [MRC Drummond] S[ain]t-Cyrille-de-Wendover, north-east, 45°57.049'N; 72°23.877'W, 22VII2003, J. Miall & P. Mason, wild radish, QC03-119 (2, CNCI); [MRC Pierre-De Saurel] S[ain]teVictoire, Hwy 239, 2km east, 45°56.580'N; 73°04.189'W, 22VII2008, ex. stem of *Raphanus raphanistrum*, em[ergence] 26VIII2008, Mason, Miall & Brauner, Sitre QC 08-710 (3, CNCI); same except: 22VII2003, 45°57.744'N; 73°06.760'W, J. Miall & P. Mason, wild radish, QC 03-114 (1 CNCI); Centre-du-Québec, [MRC Arthabaska] Saint-Rosaire, 19VII2012, swept from canola (1, CPTO); [MRC Coaticook] Compton, 27VI2014, C. Levesque (1, CNCI); same except: 24VII2014 (2, CNCI); same except: 1VIII2014 (2, CNCI).

###### Ceutorhynchus pauxillus

Taxon classificationAnimaliaColeopteraCurculionidae

Dietz, 1896, new to Quebec

Species identification confirmed by RSA, 2014, and Hiraku Yoshitake, 2014

####### Note.

This native species was previously known in Canada from Alberta, Saskatchewan, and Manitoba (Bousquet et al. 2013). The host plant for this species is unknown (Colonnelli 2004).

####### Specimen data.

[MRC de D’Autray] Lanoraie, 26VIII1986, sweeping Sphagnum bog, L. LeSage, on *Prunus pensylvanica* Linnaeus (39, CMNC; 16, CNCI); MRC Marguerite-D’Youville, Verchères, 4VI2010, C. Chantal (1, CCCH); MRC Vaudreuil-Soulanges, Saint-Lazare, 9VI2013 (15:00), sandpit, beaten from *Erysymum* sp., P. de Tonnancour (3, CPTO); same except: 12VI2013 (14:00), beaten from *Brassica* sp. (3, CPTO); same except: 14VI2013 (13:00) (1, CNCI; 1, CPTO), 19VI2013 (14:00) (9, CPTO); same except: 6VI2014 (13:00), swept from *Equisetum* and grasses (1, CPTO), 10VI2014 (17:00) (2, CPTO); same except: 23VI2014 (17:00), swept from *Equisetum* (2, CMNC; 4, CPTO).

###### Hadroplontus litura

Taxon classificationAnimaliaColeopteraCurculionidae

(Fabricius, 1775), new to Quebec

Species identification confirmed by RSA, 2016.

####### Note.

This Palaearctic species has been introduced in various parts of North America as a biological control agent against Canada thistle, *Cirsium arvense* (L.) Scop. (Asteraceae) (McClay et al. 2002), an invasive plant also of Palaearctic origin. It was previously known in Canada from British Columbia, Alberta, Saskatchewan, Ontario, and Nova Scotia (Bousquet et al. 2013). All specimens reported herein were beaten from Canada thistle.

####### Specimen data.

Montreal, Parc Zotique-Racicot (45.5427, 73.6903), beaten from *Cirsium arvense*, P. de Tonnancour and/or S. Dumont, 8VII2015 (13:00) (17, CPTO; 4, CSDU); 9VII2015 (3, CMNC; 3, CNCI; 2, CSDU); 10VII2015 (4, CCCH); 12VII2015 (16:00) (5, CPTO); 14VII2015 (15:00) (6, CPTO); 26VII2015 (2, CMNC; 2, CNCI; 2, CSDU); 25VIII2015 (1, CSDU); 01IX2015 (13:00) (1, CRVI); same except: (45.5426, 73.69O2), 28VI2016 (13:00) (7, CPTO; 2, CSDU); 30VI2016 (4, CCCH; 4 CPTO; 2, CSDU); same except: 4VII2016 (6, CSDU); MRC Vaudreuil-Soulanges, Ville de l’Île-Perrot, 11VII2016 (15:00), beaten from *Cirsium arvense*, P. de Tonnancour (1, CPTO); MRC Laval, Laval (45.5819, -73.8206), 20VII2016 (14:00), beaten from flowering *Cirsium arvense*, P. de Tonnancour (15, CPTO); Montreal, Parc Zotique-Racicot (45.5427, -73.6903), 26VII2016, beaten from *Cirsium arvense*, S. Dumont (2, CSDU); same except: 28VII2016 (4, CSDU); MRC Laval, Laval (45.5819, 73.8206), 17IX2016, beaten from flowering *Cirsium arvense*, P. de Tonnancour (5, CPTO).

###### Sirocalodes sericans

Taxon classificationAnimaliaColeopteraCurculionidae

(LeConte, 1876), new to Quebec

Species identification confirmed by Hiraku Yoshitake, 2014

####### Note.

This native species was previously known in Canada from Manitoba and Ontario (Bousquet et al. 2013), but its presence in Ontario was reported for the first time only recently (Proctor et al. 2010) based on one specimen collected in Algonquin Provincial Park in 2007. The host plant is unknown (Anderson et al. 2014).

####### Specimen data.

MRC Vaudreuil-Soulanges, Saint-Lazare, 12VI2013 (14:00), sandpit, beaten from *Brassica* sp., P. de Tonnancour (1, CPTO).

#### Subfamily Conoderinae Schönherr, 1833

##### Tribe Zygopini Lacordaire, 1865

###### Cylindrocopturus longulus

Taxon classificationAnimaliaColeopteraCurculionidae

(LeConte, 1876), new to Quebec

Species identification confirmed by Hiraku Yoshitake, 2014, and RSA, 2016.

####### Note.

This native species is reported to inhabit in the larval stage the galls formed by the apionine weevil *Podapion gallicola* Riley, 1883, on pine (Blatchley and Leng 1916). In western North America, it is also a reported host of the *Macromesus americanus* Hedqvist, 1960 (Hymenoptera: Pteromalidae), which has been reared from various pines and several other conifers (Askew and Shaw 2001). *Cylindrocopturus longulus* was previously known in Canada only from Ontario, but the gall making species *Podapion gallicola* is known from Ontario, Quebec and New Brunswick (Bousquet et al. 2013).

####### Specimen data.

MRC Vaudreuil-Soulanges, Notre-Dame-de-l’Île-Perrot, 30IV2013 (16:00), beaten from flowering shoots of *Salix* sp., P. de Tonnancour (1, CPTO); MRC Vaudreuil-Soulanges, Mont Rigaud, 31V2013 (13:00), beaten from *Asclepias syriaca*, P. de Tonnancour (1, CPTO); same except: 5VI2013 (13:00), rocky outcrop, swept from *Rumex acetosella* (1, CPTO); same except: 2V2015 (15:00), rocky outcrop, beaten from *Pinus strobus*, P. de Tonnancour (3, CPTO); MRC Collines-de-l’Outaouais, Luskville (Sentier des chutes), 26V2015 (13:00), beaten from small *Amelanchier* sp., P. de Tonnancour (1, CPTO).

#### Subfamily Entiminae Schönherr, 1823

##### Tribe Cyphicerini Lacordaire, 1863

###### Myosides seriehispidus

Taxon classificationAnimaliaColeopteraCurculionidae

Roelofs, 1873, new to Quebec

Species identification confirmed by RSA, 2016

####### Note.

This adventive species, originally from Asia, has gone undetected for many years in collections under the genus *Trachyphloeus* Germar, 1817, and was reported by O’Brien (2000) as established in several eastern states since at least 1973. In Canada, it was known until now only from Ontario (Bousquet et al. 2013). Only females are known to occur in North America (Bright and Bouchard 2008).

####### Specimen data.

[MRC Brome-Missisquoi], Saint-Armand, 6VII2015 (afternoon), C. Chantal (1, CCCH).

#### Subfamily Hyperinae Marseul, 1863

##### Tribe Hyperini Marseul, 1863

###### Hypera rumicis

Taxon classificationAnimaliaColeopteraCurculionidae

(Linnaeus, 1758), new to Quebec

Species identification confirmed by Hiraku Yoshitake, 2014

####### Note.

Since its accidental introduction and first detection in the United States in 1879 (Chamberlin, 1933), this Palaearctic species has expanded its range considerably in North America. Surveys conducted from 1997 to 1999 in two Quebec vineyards failed to detect its presence (Bouchard et al. 2005), but its capture by C. Tessier in 2003 indicates that it was already present in the province more than a decade ago. *Hypera rumicis* is associated with various *Polygonum* L. spp. and *Rumex* spp. (Polygonaceae), especially the invasive curled dock, *Rumex crispus* L., also introduced from Europe. Its potential as a biological control agent against this weed was recently assessed (DeGregorio et al. 1992; Piesik 2006). This species was previously known in Canada from Alberta, Saskatchewan, Manitoba, and Ontario (Bousquet et al. 2013).

####### Specimen data.

[MRC Brome-Missisquoi], Saint-Armand, 15VI2003, C. Tessier (1, CCTE); MRC Vaudreuil-Soulanges, Notre-Dame-de-l’Île-Perrot, 3VII2011 (17:00), beaten from *Rumex crispus*, P. de Tonnancour (1, CPTO); MRC Vaudreuil-Soulanges, Terrasse-Vaudreuil, 6VII2011 (2:00), UV + porch light, P. de Tonnancour (1, CPTO); MRC Haut-Saint-Laurent, Saint-Anicet (45.0425, -74°4459), 15VI2013 (13:00), wet meadow, swept from various herbaceous plants, P. de Tonnancour (2, CPTO); Montreal, Île-Bizard (Parc-nature du Bois-de-l’Île-Bizard), 17VI2013, ≥ 5 cocoons on *Rumex* sp. (one emergence on 22VI2013), C. Pilon (observation documented by photos); MRC Haut-Saint-Laurent, Saint-Anicet (45.0432, -74°4442), 26VI2015 (15:00), beaten from *Rumex crispus*, P. de Tonnancour (11, CPTO); MRC Coaticook, Waterville (45.27993 N 71.89987 O), 10VII2015 (20:00), beaten from *Rumex crispus*, P. de Tonnancour (4, CCCH; 7, CMNC; 2, CSDU; 1, CRVI); same except 11VII2015 (10:00), H. Miquet-Sage, P. de Tonnancour, S. Dumont (7, CHMS; 18, CPTO; 7, CSDU, 1, CRVI); MRC Haut-Richelieu, Henryville, dike adjacent to Réserve écologique Marcel-Raymond, 12V2016 (13:00–16:00), swept from grasses, *Equisetum* and *Solidago*, P. de Tonnancour 12V2016 (1, CPTO).

#### Subfamily Lixinae Schönherr, 1823

##### Tribe Lixini Schönherr, 1823

###### Lixus punctinasus

Taxon classificationAnimaliaColeopteraCurculionidae

LeConte, 1876, first records for Canada with detailed locality information

Species identification confirmed by RSA, 2015

####### Note.

Bousquet et al. (2013) listed this species without providing any details on its distribution in Canada, based on a similar inclusion in O’Brien and Wibmer (1982). We provide specific locality data for Canada for the first time. Like other *Lixus* Fabricius, 1801 spp., this species is probably associated with *Polygonum* spp. (Polygonaceae). Numerous CMNC specimens from Texas were collected on *Polygonum*.

####### Specimen data.

MRC Vaudreuil-Soulanges, Notre-Dame-de-l’Île-Perrot, 3VII2008 (17:00), handpicked from building wall, P. de Tonnancour (1, CPTO); same except: 23VIII2014 (15:00), small pond margin, beaten from *Bidens cernua*, P. de Tonnancour (1, CPTO).

###### Lixus terminalis

Taxon classificationAnimaliaColeopteraCurculionidae

LeConte, 1876, new to Quebec

Species identification confirmed by RSA, 2015

####### Note.

This species was reported to be associated with *Polygonum amphibium* L. (now *Persicaria amphibia* (L.) Delarbre) (Polygonaceae) more than a century ago (Beutenmuller 1893). We also found it to be common on this same species of plant in Oka.

####### Specimen data.

[MRC Deux-Montagnes] Parc national d’Oka, 4V1993, R. Vigneault (1, CRVI); same except: 9V1993 (1, CRVI), 30V1995 (1, CRVI); [MRC Deux-Montagnes] Parc national d’Oka, 26V2002, flowers of *Prunus virginiana*, C. Chantal (1, CCCH); [MRC Deux-Montagnes] Parc national d’Oka, La Grande Baie, 28V2002, R. Vigneault (1, CRVI); same except: 4V2003 (1, CRVI); [MRC Deux-Montagnes] Parc national d’Oka, Calvaire d’Oka, 15VII2007, R. Vigneault (1, CRVI); [MRC Deux-Montagnes] Parc national d’Oka, 16VI2011, R. Vigneault (1, CRVI); [MRC Deux-Montagnes] Parc national d’Oka, 1VIII2012 (16:00–17:00), swept from *Polygonum* sp., P. de Tonnancour & R. Vigneault (5, CPTO; 2, CRVI); same except: 19VIII2012 (17:00), swept from *Polygonum amphibium*, P. de Tonnancour (2, CMNC; 21, CPTO); same except: 26VIII2012 (17:00) (4, CPTO); same except: 18V2013 (15:00), beaten from *Crataegus* sp., P. de Tonnancour (1, CPTO); same except: 25V2014, composting site, white tulle fabric flight interception trap, R. Vigneault (1, CRVI).

#### Subfamily Molytinae Schönherr, 1823

##### Tribe Cleogonini Gistel, 1848

###### Rhyssomatus aequalis

Taxon classificationAnimaliaColeopteraCurculionidae

Horn, 1873, first records for Quebec with detailed locality information

Species identification confirmed by RSA, 2015

####### Note.

This native species was known in Canada only from Ontario (McNamara 1991) until Bousquet et al. (2013) recorded it from Quebec without providing any specific details about its distribution within the province. Before the recent addition of a few specimens from the series reported herein, the CNCI contained 33 specimens from Canada, all collected by W.J. Brown in extreme southern Ontario between 1931 and 1940, 17 of them on “*Convolvulus sepium pubescens*” (hedge false bindweed, now known as *Calystegia sepium* (L.) R. Br. (Convolvulaceae)). Based on these label data and on those of most specimens reported henceforth, *R. aequalis* appears to be associated with *C. sepium*.

####### Specimen data.

MRC Haut-Saint-Laurent, Saint-Anicet (45.0425, -74°4459), 15VI2013, wet meadow, swept from various herbaceous plants, P. de Tonnancour & S. Laplante (1, CPTO; 2, CSLA); Montreal, Parc Zotique-Racicot (45.5436, 73.69O3), 8VII2015 (13:00), beaten from *Castylegia sepium* + *Cirsium arvense*, P. de Tonnancour (1, CPTO); same except: 9VII2015, S. Dumont (2, CSDU), 24VII2015 (4, CPTO); same except: 26VII2105, beaten from *Castylegia sepium* + *Cirsium arvense*, S. Dumont (1, CMNC; 1, CNCI; 1, CSDU); same except: 25VIII2015, beaten from *Castylegia sepium* + *Cirsium arvense*, S. Dumont (2, CPTO; 2, CSDU); same except: 1IX2015 (13:00), beaten from *Castylegia sepium*, P. de Tonnancour & S. Dumont (1, CMNC; 1, CNCI; 6, CPTO; 1, CSDU); same except: 7VI2016 S. Dumont (13, CSDU; 10, CCCH); same except: 10VI2106 (8, CSDU); 14VI21016 (11, CSDU); MRC Vaudreuil-Soulanges, Ville-de-l’Île-Perrot, 15VI2016 (12:00), beaten from *Castylegia sepium*, P. de Tonnancour (2, CPTO); Montreal, Parc Zotique-Racicot (45.5436, 73.69O3), 16VI2016, beaten from *Castylegia sepium*, S. Dumont (1, CSDU); MRC Vaudreuil-Soulanges, Notre-Dame-de-l’Île-Perrot, 17VI2016 (12:30), beaten from *Castylegia sepium*, P. de Tonnancour (3, CPTO); MRC Vaudreuil-Soulanges, Ville-de-l’Île-Perrot (45.3970, -73.9629), 18VI2016 (12:00), beaten from *Castylegia sepium*, P. de Tonnancour (1, CPTO); MRC Vaudreuil-Soulanges, Notre-Dame-de-l’Île-Perrot, 21VI2016 (17:00), beaten from *Castylegia sepium*, P. de Tonnancour (4, CPTO); Montreal, Parc Zotique-Racicot (45.5436, 73.69O3), 23VI2016, beaten from *Castylegia sepium*, S. Dumont (1, CSDU); same except: 28VI2015 (13:00), P. de Tonnancour (14, CPTO); same except: 30VI2016, S. Dumont (2, CSDU); same except: 4VII2016 (2, CSDU); same except: 26VII2016 (2, CSDU); same except: 28VII2016 (1, CSDU); same except: 18VIII2016 (1, CSDU).

##### Tribe Conotrachelini Jekel, 1865

###### Conotrachelus buchanani

Taxon classificationAnimaliaColeopteraCurculionidae

Schoof, 1942, new to Canada

Species identification confirmed by RSA, 2015

####### Note.

This native species is associated with *Celtis* L. spp. (Cannabaceae) (Schoof 1942), specifically common hackberry, *Celtis occidentalis* L., in Quebec. All specimens collected in Montreal were beaten from common hackberry, and all those from Terrasse-Vaudreuil were attracted to a light source located no more than 10 m from a hackberry tree. Hackberry has been favoured as a street tree and planted in great numbers in some boroughs by the City of Montreal between 1972 and 1984 (QuéBio 2016), obviously much to the benefit of the weevil. Records provided herein represent a significant northerly extension of the range of this species which was previously only known from as far north as Pennsylvania (O’Brien and Wibmer 1982).

Specimens from southern USA were examined and found to be consistently larger than the northern forms from Quebec and northern USA, but dissections failed to reveal any further significant differences between the two groups. The status of the Canadian and northern USA forms needs further study. For the time being specimens reported herein will be considered as *C. buchanani*.

####### Specimen data.

[MRC Brome-Missisquoi], Saint-Armand, 5VI2007, C. Chantal (1, CCCH); MRC Vaudreuil-Soulanges, Terrasse-Vaudreuil, 21V2009 (21:00–22:00), mercury vapour light, P. de Tonnancour (1, CPTO); same except: 18VI2010 (23:00), mercury vapour + UV + porch light, P. de Tonnancour (1, CPTO); same except: 6VII2011 (23:00), UV + porch light, P. de Tonnancour (1, CPTO); MRC Marguerite-D’Youville, Contrecœur, 8VII2012 (0:30), mercury vapour + UV light, P. de Tonnancour (1, CPTO); [MRC La ValléeduRichelieu] MontSaintHilaire, 2-VI-2013, H. Miquet-Sage (2, CHMS); MRC Vaudreuil-Soulanges, Terrasse-Vaudreuil, 21IX2014 (21:00), UV + porch light, P. de Tonnancour (1, CPTO); Montreal, Parc Zotique-Racicot (45.5424, 73.6874), 19VI2015, beaten from *Celtis occidentalis*, S. Dumont (3, CMNC; 3, CNCI; 3, CSDU); same except: 2VII2015 (5, CSDU); Montreal, 11875, rue Zotique-Racicot (45.5424, -73.6901), beaten from *Celtis occidentalis*, 8VII2015, P. de Tonnancour & S. Dumont (6, CPTO; 1, CSDU); same except: 12VII2015 (16:00), P. de Tonnancour (2, CPTO); same except: 9VII2015, S. Dumont (2, CMNC; 2, CNCI; 1, CSDU); same except: 16VIII2015 (6, CMNC; 6, CNCI; 1, CSDU); 21VIII2015 (2, CSDU); 25-VIII-2015 (3, CSDU); same except: 1IX2015, P. de Tonnancour & S. Dumont (21, CPTO; 2, CSDU); same except: 12X2015, S. Dumont (2, CSDU); same except: 22V2016 (3, CSDU); same except: 24V2016 (5, CSDU); MRC Vaudreuil-Soulanges, Terrasse-Vaudreuil, 30V2016 (01:00), UV + porch light, P. de Tonnancour (1, CPTO); Montreal, Parc Zotique-Racicot (45.5424, -73.6874), 7VI2016, beaten from *Celtis occidentalis*, S. Dumont (9, CSDU); same except: 14VI2016 (1, CSDU); same except: 27VI2016, UV light (1, CSDU); MRC Vaudreuil-Soulanges, Terrasse-Vaudreuil, 27VI2016 (22:45), UV + porch light, P. de Tonnancour (1, CPTO); Montreal, Parc Zotique-Racicot (45.5424, -73.6874), 30VI2016, beaten from *Celtis occidentalis*, S. Dumont (10, CCCH; 7, CSDU); same except: 8VIII2016 (13, CSDU); same except: 18VIII2016 (3, CSDU).

###### Conotrachelus pusillus

Taxon classificationAnimaliaColeopteraCurculionidae

LeConte, 1878, new to Canada

Species identification confirmed by RSA, 2015, 2016

####### Note.

This native species was previously known to occur in eastern North America from New York and Florida to Kansas and Texas (O’Brien and Wibmer 1982). Host plants are unknown.

####### Specimen data.

[MRC Deux-Montagnes] Parc national d’Oka, composting site, 23VII2011, R. Vigneault (1, CRVI); same except: plage d’Oka, 2VIII2011 (1, CRVI); [MRC Marguerite-D’Youville] Varennes, 8IX2015, attracted to UV lamp, C. Chantal (1, CCCH); MRC Deux-Montagnes, Parc national d’Oka, 21VII2015 (1:00), beaten from foliage of *Carya ovata*, P. de Tonnancour (1, CPTO).

###### Conotrachelus recessus

Taxon classificationAnimaliaColeopteraCurculionidae

(Casey, 1910), new to Quebec

Species identification confirmed by RSA, 2015

####### Note.

This small *Conotrachelus* Dejean, 1835 was previously known in Canada only from Ontario (Bousquet et al. 2013). It is superficially similar to a *Tychius* Germar, 1817 sp. and was in fact originally described by Casey in the tribe Tychiini (Curculioninae) as the type of the monobasic genus *Loceptes* Casey, 1910 (Schoof 1942). It can be separated from its congeners by its very small size (2.5 – 3.0mm), golden colored scales and recurved elytral setae. Available data on host plants are inconclusive.

####### Specimen data.

MRC Vaudreuil-Soulanges, Terrasse-Vaudreuil, 19VI2014 (0:00), attracted to UV + porch light, P. de Tonnancour (1, CPTO).

#### Subfamily Scolytinae Latreille, 1804

##### Tribe Phloeotribini Chapuis, 1869

###### Phloeotribus dentifrons

Taxon classificationAnimaliaColeopteraCurculionidae

(Blackman, 1921), new to Quebec

Species identification confirmed by Hume Douglas, 2016

####### Note.

This minute native species (1.2–1.6mm) was previously known to occur in Canada only in the southernmost part of Ontario (all 61 CNCI Canadian specimens are from Point Pelee National Park). As for the above-mentioned *Conotrachelus buchanani*, this native species is associated with *Celtis* spp. (Wood 1982), specifically common hackberry, *Celtis occidentalis*, in Quebec. It is probably more widely distributed than currently known in Quebec, as it was found in close association with its host plant in three different localities. It has also been reared recently (2016) from dead branches of *Celtis occidentalis* in Almonte, Ontario, ca. 30 km from the Quebec border (Hume Douglas, pers. comm. 2017).

####### Specimen data.

MRC Vaudreuil-Soulanges, Terrasse-Vaudreuil (45.3923, 73.9922), 20IX2013 (18:00), white tulle fabric flight interception trap, P. de Tonnancour (1, CPTO); same except: 20V2016 (16:30) (1, CPTO); Montreal, rue Zotique-Racicot (45.5436, -73.6901), 21VIII2015, beaten from *Celtis occidentalis*, S. Dumont (1, CSDU); same except: 21V2016 (9, CSDU); same except: 22V2016 (4, CSDU); same except: 23V2016 (7, CPTO); same except: 24V2016 (3, CSDU); same except: 7VI2016 (1, CSDU); MRC Laval, Laval, rue des Charmes (45.5888, 73.8268), 20VII2016 (15:00), beaten from *Celtis occidentalis*, P. de Tonnancour (1, CPTO); MRC Vaudreuil-Soulanges, Terrasse-Vaudreuil (45.3927, -73.9922), 20VII2016, ex-larva from dead branch of *Celtis occidentalis*, P. de Tonnancour (3, CPTO); same except: 28-VII-2016 (1, CPTO); same except: 14-VIII-2016 (2, CPTO); same except: 15-VIII-2016 (1, CPTO); same except : 18-III-2017 (8, CNCI); MRC Laval, Laval, rue des Charmes (45.5846, 73.8226), 2IV2017, ex-larva from dead branch of *Celtis occidentalis*, P. de Tonnancour (5, CPTO).

##### Tribe Scolytini Latreille, 1804

###### Scolytus muticus

Taxon classificationAnimaliaColeopteraCurculionidae

Say, 1824, new to Quebec

Species identification confirmed by Hume Douglas, 2016

####### Note.

As for the above-mentioned *Phloeotribus dentifrons*, this native species was previously thought to be confined in Canada to the southernmost part of Ontario (all 11 CNCI Canadian specimens are from Pelee Island and Point Pelee National Park). It occurs in association with common hackberry, *Celtis occidentalis*, in Quebec, but also with dwarf hackberry, *Celtis tenuifolia* Nutt. (Smith and Cognato 2014), an endangered species, in southern Ontario (COSEWIC 2003). At 2.2–5.3mm, it is the largest member of the genus known to occur in Quebec.

####### Specimen data.

MRC Vaudreuil-Soulanges, Terrasse-Vaudreuil, 14VI2016 (14:00), white tulle fabric flight interception trap, P. de Tonnancour (1, CPTO); MRC Laval, Laval, rue des Charmes (45.5846, -73.8226), 6VII2016 (15:00), beaten from *Celtis occidentalis*, P. de Tonnancour (1, CPTO).

## Supplementary Material

XML Treatment for Choragus sayi

XML Treatment for Perapion punctinasum

XML Treatment for Omphalapion hookerorum

XML Treatment for Ischnopterapion virens

XML Treatment for Coelocephalapion emaciipes

XML Treatment for Anthonomus robustulus

XML Treatment for Pseudanthonomus helvolus

XML Treatment for Curculio rubidus

XML Treatment for Smicronyx sculpticollis

XML Treatment for Bagous magister

XML Treatment for Bagous tanneri

XML Treatment for Buchananius striatus

XML Treatment for Plocamus echidna

XML Treatment for Ceutorhynchus bolteri

XML Treatment for Ceutorhynchus pallidactylus

XML Treatment for Ceutorhynchus pauxillus

XML Treatment for Hadroplontus litura

XML Treatment for Sirocalodes sericans

XML Treatment for Cylindrocopturus longulus

XML Treatment for Myosides seriehispidus

XML Treatment for Hypera rumicis

XML Treatment for Lixus punctinasus

XML Treatment for Lixus terminalis

XML Treatment for Rhyssomatus aequalis

XML Treatment for Conotrachelus buchanani

XML Treatment for Conotrachelus pusillus

XML Treatment for Conotrachelus recessus

XML Treatment for Phloeotribus dentifrons

XML Treatment for Scolytus muticus

## References

[B1] Alonso-Zarazaga MA (2011) Family Apionidae Schoenherr, 1823. In: Löbl I Smetana A (Eds) Catalogue of Palaearctic Coleoptera. Volume 7. Curculionoidea I. Apollo Books, Stenstrup, 148–176.

[B2] Anderson DM (1962) The weevil genus *Smicronyx* in America north of Mexico (Coleoptera: Curculionidae). Proceedings of the United States National Museum 113: 185–372. https://doi.org/10.5479/si.00963801.113-3456.185

[B3] Anderson RS Bouchard P Douglas H (2014) Weevils (Coleoptera: Dryophthoridae, Brachyceridae, Curculionidae) of the Prairie Ecozone in Canada. In: Giberson DJ Carcámo HA (Eds) Arthropods of Canadian Grasslands. Volume 4: Biodiversity and Systematics. Part 2. Biological Survey of Canada, 143–167.

[B4] Anderson RS Keller O Prena J (2013) *Curculio rubidus* Gyllenhal, 1836 (Coleoptera: Curculionidae), a European weevil new to North America. The Coleopterists Bulletin 67(3): 368–369. https://doi.org/10.1649/0010-065X-67.3.368

[B5] Askew RR Shaw MR (2001) An annotated list of *Macromesus* Walker and a British record for *M. amphiretus* Walker (Hym., Pteromalidae). Entomologist’s Monthly Magazine 137(1648): 227–231.

[B6] Beutenmuller W (1893) On the food-habits of North American Rhynchophora. Journal of the New York Entomological Society 1: 36–43.

[B7] Blatchley WS Leng CW (1916) Rhynchophora or Weevils of North Eastern America. The Nature Publishing Company, Indianapolis, 682 pp https://doi.org/10.5962/bhl.title.1557

[B8] Bouchard P Bousquet Y Davies AE Alonso-Zarazaga MA Lawrence JF Lyal CHC Newton AF Reid CAM Schmitt M Ślipiński SA Smith ABT (2011) Family-group names in Coleoptera (Insecta). ZooKeys 88: 1–972. https://doi.org/10.3897/zookeys.88.807 10.3897/zookeys.88.807 PMC3088472 21594053

[B9] Bouchard P LeSage L Goulet H Bostanian NJ Vincent C Zmudzinska A Lasnier J (2005) Weevil (Coleoptera: Curculionoidea) diversity and abundance in two Quebec vineyards. Annals of the Entomological Society of America 98: 565–574. https://doi.org/10.1603/0013-8746(2005)098[0565:WCCDAA]2.0.CO;2

[B10] Bousquet Y Bouchard P Davies AE Sikes DS (2013) Checklist of Beetles of Canada and Alaska. Second edition. Pensoft, Sofia, 402 pp. 10.3897/zookeys.360.4742 PMC3867111 24363590

[B11] Bright DE (1993) The insects and arachnids of Canada. Part 21. The Weevils of Canada and Alaska. Volume 1. Coleoptera: Curculionoidea, excluding Scolytidae and Curculionidae. Agriculture Canada Publication 1882, Ottawa, 217 pp.

[B12] Bright DE Bouchard P (2008) Weevils of Canada and Alaska. Volume 2 Coleoptera: Curculionidae, Entiminae. NRC Research Press, Ottawa, i-xiii + 327 pp.

[B13] Chamberlin TR (1933) Some observations on the life history and parasites of *Hypera rumicis* (L.) (Coleoptera: Curculionidae). Proceedings of the Entomological Society of Washington 35(6): 101–109.

[B14] Clark WE (1987) Revision of the Nearctic species of *Pseudanthonomus* Dietz (Coleoptera: Curculionidae). The Coleopterists Bulletin 41: 263–285.

[B15] Colonnelli E (2004) Catalogue of the Ceutorhynchinae of the World with a Key to the Genera. Argania Editio, Barcelona, 124 pp.

[B16] COSEWIC (2003) COSEWIC assessment and update status report on the dwarf hackberry *Celtis tenuifolia* in Canada. Committee on the Status of Endangered Wildlife in Canada, Ottawa, vi + 15 pp. www.sararegistry.gc.ca/status/status_e.cfm

[B17] DeGregorio RE Ashley RA Streams FA Adams RG Jr Schaefer CW (1992) Biocontrol potential of *Hypera rumicis* (L.) (Coleoptera: Curculionidae) on curly dock (*Rumex crispus* L.). Journal of Sustainable Agriculture 2(1): 7–24. https://doi.org/10.1300/J064v02n01_03

[B18] DeSteven D (1981) Abundance and survival of a seed infesting weevil, *Pseudanthonomus hamamelidis* (Coleoptera: Curculionidae) on its variablefruiting host plant, witch hazel (*Hamamelis virginiana*). Ecological Entomology 6(4): 387–396. https://doi.org/10.1111/j.1365-2311.1981.tb00629.x

[B19] Douglas H Bouchard P Anderson RS de Tonnancour P Vigneault R Webster RP (2013) New Curculionoidea (Coleoptera) records for Canada. Zookeys 309: 13–48. https://doi.org/10.3897/zookeys.309.4667 10.3897/zookeys.309.4667 PMC3689125 23794927

[B20] Hoebeke ER Byers RA Alonso-Zarazaga MA Stimmel JF (2000) Ischnopterapion (Chlorapion) virens (Herbst) (Coleoptera: Curculionoidea: Brentidae: Apioninae), a Palearctic clover pest new to North America: Recognition features, distribution, and bionomics. Proceedings of the Entomological Society of Washington 102(1): 151–161.

[B21] Hoffmann A (1954) Coléoptères Curculionides (Deuxième Partie). 59 Faune de France. Fédération Française des Sociétés de Sciences Naturelles, Librairie de la Faculté des sciences, Paris, France.

[B22] Majka CG Anderson RS McCorquodale DB (2007a) The weevils (Coleoptera: Curculionoidea) of the Maritime Provinces of Canada, II: New records from Nova Scotia and Prince Edward Island and regional zoogeography. The Canadian Entomologist 139: 397–442.

[B23] Majka CG Anderson RS Georgeson E (2007b) Introduced Apionidae and Brentidae (Coleoptera: Curculionoidea) in the Maritime Provinces of Canada. Proceedings of the Entomological Society of Washington 109(1): 66–74.

[B24] McClay A De Clerck-Floate R (1999) Establishment and early effects of *Omphalapion hookeri* (Kirby) (Coleoptera: Apionidae) as a biological control agent for scentless chamomile, *Matricaria perforata* Mérat (Asteraceae). Biological Control 14: 85–95. https://doi.org/10.1006/bcon.1998.0679

[B25] McClay AS Bourchier RS Butts RA Peschken DP (2002) 65. *Cirsium arvense* (L.) Scopoli, Canada thistle (Asteraceae). In: Mason PG Huber JT (Eds) Biological control programmes in Canada, 1981–2000. CABI Publishing, Wallingford, 318–330.

[B26] McGaha YJ (1952) The limnological relations of insects to certain aquatic flowering plants. Transactions of the American Microscopical Society 71: 355–381. https://doi.org/10.2307/3223467

[B27] McNamara J (1991) Superfamily Curculionoidea. In: Bousquet Y (Ed.) Checklist of the Beetles of Canada and Alaska. Agriculture Canada, Ottawa, 323–365.

[B28] O’Brien CW (2000) *Myosides seriehispidus* Roelofs, an Asian weevil new to the United States (Coleoptera, Curculionidae). Insecta Mundi 14: 229–231.

[B29] O’Brien CW Marshall GB (1979) U.S. *Bagous*, bionomic notes, a new species, and a new name (Bagoini, Erirhininae, Curculionidae, Coleoptera). The Southwestern Entomologist 4(2): 141–149.

[B30] O’Brien CW Wibmer GJ (1982) Annotated checklist of the weevils (Curculionidae sensu lato) of North America, Central America, and the West Indies (Coleoptera: Curculionoidea). Memoirs of the American Entomological Institute 34: ix + 1–382.

[B31] Peschken DP Sawchyn KC Bright DE (1993) First record of *Apion hookeri* Kirby (Coleoptera: Curculionidae) in North America. The Canadian Entomologist 125: 629–631. https://doi.org/10.4039/Ent125629-3

[B32] Piesik D (2006) Impact of herbicide on mossy sorrel (*Rumex confertus*), and phytophagous *Hypera rumicis*, *Apion miniatum* and *Pegomya nigritarsis*. Electronic Journal of Polish Agricultural Universities, Biology 9(2), article 23.

[B33] Prena J Steiner WE Jr Grebennikov VV (2014) *Buchananius sulcatus* (LeConte) (Coleoptera: Curculionidae: Baridinae) reared from the fruiting bodies of the Ascomycete fungus *Trichoderma peltatum* (Berk.) Samuels, Jaklitsch, and Voglmayr in Maryland, USA. The Coleopterists Bulletin 68: 399–402. https://doi.org/10.1649/072.068.0310

[B34] Proctor E Anderson RS Nol E Girard JM Richmond S (2010) Ground-dwelling weevil (Coleoptera: Curculionidae) communities in fragmented and continuous hardwood forests in south-central Ontario. Journal of the Entomological Society of Ontario 141: 69–83.

[B35] QuéBio (2016) Public trees of Montreal. http://www.quebio.ca/en/arbresmtl [accessed April 20, 2016]

[B36] Sampson MG MacSween T (1993) Biological control of weeds in Nova Scotia. Nova Scotia Department of Agriculture and Marketing, Final Report TD63, 47 pp.

[B37] Schoof HF (1942) The genus *Conotrachelus* Dejean (Coleoptera: Curculionidae) in the North Central United States. Contribution to the Entomological Laboratories of the University of Illinois, University of Illinois Press, 170 pp.

[B38] Smith SM Cognato AI (2014) A taxonomic revision of Nearctic *Scolytus* Geoffroy (Coleoptera, Curculionidae, Scolytinae). ZooKeys 450: 1–182. https://doi.org/10.3897/zookeys.450.7452 10.3897/zookeys.450.7452 PMC4233402 25408617

[B39] Tanner VM (1943) A study of the subtribe Hydronomi with a description of a new species, (Curculionidae). Study No. VI. Great Basin Naturalist 4: 1–38.

[B40] Valentine BD (1998) A review of Nearctic and some related Anthribidae (Coleoptera). Insecta Mundi 12(3-4): 251–296.

[B41] Wheeler AG Jr Hoebeke ER (2009) Chapter 21. Adventive (non-native) insects: importance to science and society. In: Foottit RG Adler PH (Eds) Insect Biodiversity: Science and Society. Blackwell Publishing, New Jersey, 475–522. https://doi.org/10.1002/9781444308211.ch21

[B42] Wood SL (1982) The bark and ambrosia beetles of North and Central America (Coleoptera: Scolytidae), a taxonomic monograph. The Great Basin Naturalist Memoirs No 6, 1359 pp.

